# Treatment and imaging of intracranial atherosclerotic stenosis: current perspectives and future directions

**DOI:** 10.1002/brb3.536

**Published:** 2016-08-31

**Authors:** Ido R. van den Wijngaard, Ghislaine Holswilder, Marianne A. A. van Walderveen, Ale Algra, Marieke J. H. Wermer, Osama O. Zaidat, Jelis Boiten

**Affiliations:** ^1^ Department of RadiologyLeiden University Medical CenterLeidenthe Netherlands; ^2^ Department of NeurologyMedical Center Haaglandenthe Haguethe Netherlands; ^3^ Department of Clinical EpidemiologyLeiden University Medical CenterLeidenthe Netherlands; ^4^ Department of Neurology and NeurosurgeryBrain Center Rudolf MagnusUniversity Medical Center UtrechtUtrechtthe Netherlands; ^5^ Department of NeurologyLeiden University Medical CenterLeidenthe Netherlands; ^6^ Department of NeurologyMedical College of Wisconsin/Froedtert HospitalMilwaukeeWIUSA

**Keywords:** intracranial atherosclerosis, intracranial stenosis, ischemic stroke

## Abstract

**Background and Purpose:**

Intracranial atherosclerosis is a common cause of stroke worldwide. It results in ischemic stroke due to different mechanisms including artery‐to‐artery embolism, in situ thrombo‐occlusion, occlusion of perforating arteries, and hemodynamic failure. In this review, we present an overview of current treatment and imaging modalities in intracranial atherosclerotic stenosis.

**Methods:**

PubMed was searched for relevant articles in English that evaluated the treatment and imaging of intracranial atherosclerotic stenosis (ICAS).

**Results:**

Aggressive medical management, consisting of dual antiplatelet therapy and intensive risk factor management, is important in patients with ICAS because of a substantial risk of recurrent stroke, approximately 20% in the first year, in patients on aspirin or warfarin alone. Recent trials have suggested that, aggressive medical therapy results in better outcome as compared with intracranial stenting. However, the question remains what the optimal treatment strategy would be in patients with recurrent strokes in the setting of failed aggressive medical therapy. Moreover, controversy exists whether a subgroup of patients with symptomatic ICAS could benefit from intracranial stenting if selection is based on radiological evidence of hemodynamic failure. With regard to imaging, transcranial Doppler ultrasound and magnetic resonance angiography are useful screening tests for exclusion of ICAS, but need confirmation by other imaging modalities when stenosis is suggested. Computed tomography angiography has a high positive and negative predictive value for detection of intracranial luminal stenosis of 50% or higher, but performs worse than digital subtraction angiography with regard to establishing the exact degree of luminal stenosis. Novel imaging techniques including high‐resolution CT and MRI better identify plaque characteristics than conventional imaging methods.

**Conclusions:**

Currently, aggressive medical management remains the standard of care for patients with ICAS. Further research is needed to identify high‐risk subgroups and to develop more effective treatments for ICAS patients.

## 1 Introduction

The global burden of stroke is large and increasing (Feigin et al., 2014). Cardioembolic stroke, large artery atherosclerosis, and small vessel disease are the most common subtypes encountered in clinical practice. Also, a combination of these subtypes, that is, vascular pathology at multiple sites, is present in many ischemic stroke patients (Bos et al., 2014). Recently, intracranial atherosclerosis has been suggested to be the most common cause of stroke worldwide (Arenillas, 2011; Gorelick, Wong, Bae, & Pandey, 2008). Racial differences in the distribution of atherosclerosis have long been a subject of interest. Caucasians have a higher incidence rate of extracranial atherosclerosis, whereas African‐Americans and Asians have a higher rate of intracranial atherosclerosis (Leung, Ng, Yuen, Lauder, & Ho, 1993; Solberg et al., 1968). Recently, however, intracranial atherosclerosis was shown to be a more important risk factor for ischemic stroke in Caucasians than reported previously (Bos et al., 2012, 2014; Mazighi et al., 2008). The most common sites for intracranial atherosclerosis are the internal carotid artery (ICA), middle cerebral artery (MCA), and basilar artery (BA). The intracranial vertebral artery (VA), posterior and anterior cerebral arteries (PCA and ACA) are less commonly affected, whereas cerebellar and communicating arteries are rarely involved (van der Kolk, Zwanenburg, Brundel, et al., 2015; Resch, Loewenson, & Baker, 1970; Wityk et al., 1996).

Intracranial atherosclerotic stenosis (ICAS) represents a more advanced stage of intracranial atherosclerotic disease, in which the vessel lumen has narrowed, in clinical practice usually 50–99% of the diameter, due to atherosclerotic lesions (Qureshi et al., 2009). In asymptomatic individuals, the prevalence of ICAS has been estimated around 4 to 13% depending on age and ethnicity (Suri & Johnston, 2009). ICAS is considered to be a major cause of stroke in the Asian, African‐American, and Hispanic populations (Liu, Tu, Yip, & Su, 1996; Sacco, Kargman, Gu, & Zamanillo, 1995; Wang et al., 2014; White et al., 2005), whereas ICAS causes stroke in only about 10% of Caucasians (Gorelick et al., 2008; Sacco et al., 1995). The estimated prevalence of symptomatic ICAS in more recent literature ranges from 20% to 53%, depending on the study population, race, and imaging method (Ritz, Denswil, Stam, van Lieshout, & Daemen, 2014).

Patients with symptomatic ICAS are at high risk of recurrent stroke of up to 25–30% in 2 years after the initial event (Kasner et al., 2006; Wong, 2006). ICAS is also associated with depression, cognitive deficits, and dementia (Qureshi & Caplan, 2014; Yarchoan et al., 2012). Despite the high prevalence of this high‐risk disease (Chimowitz et al., 2005; Gorelick et al., 2008; Mazighi et al., 2006, 2008), controversy exists regarding treatment of symptomatic ICAS patients, in particular with regard to endovascular treatment of certain high‐risk subgroups of patients.

Moreover, uncertainty exists about the most optimal noninvasive imaging modality to evaluate presence, extent, and stroke risk of intracranial atherosclerosis. In this review, we first will discuss different stroke mechanisms and prognostic radiological features of ICAS which might influence imaging and treatment strategies. Subsequently, we will give an update on treatment options and discuss controversies in treatment of ICAS. Also, we will review different imaging modalities available for the evaluation of intracranial atherosclerotic plaques and stenosis, with an emphasis on their predictive values and use in clinical practice. Finally, new noninvasive imaging techniques capable of imaging the arterial wall of intracranial arteries will be discussed.

### 1.1 Search strategy

The references for this narrative review were identified through searches of the PubMed database up to October 2015. PubMed was first searched for articles on intracranial atherosclerosis/stenosis and subsequently for imaging studies of intracranial atherosclerosis/stenosis. The search items “intracranial stenosis” OR “intracranial atherosclerosis” AND “imaging” OR “treatment” were combined. In addition, this search was extended with the combination of “intracranial stenosis” or “intracranial atherosclerosis” AND the different imaging techniques separately: “digital subtraction angiography” (DSA), “transcranial Doppler ultrasound” (TCD), “computed tomography” (CT), “computed tomography angiography” (CTA), “magnetic resonance angiography” (MRA), “high resolution imaging”, “vessel wall imaging” (VWI). Only papers written in English were considered. Additional papers were identified through searches based on the references of relevant studies.

## 2 Stroke Mechanisms in Intracranial Atherosclerotic Stenosis

The underlying mechanism of ischemic stroke in ICAS is typically inferred by infarct pattern on neuroimaging (Holmstedt, Turan, & Chimowitz, 2013). Typical patterns are border zone infarctions as a result of hypoperfusion due to a highly stenotic artery, and territorial infarctions as a result of artery‐to‐artery embolism. One of the possible causes of a lacunar infarction in ICAS is plaque extension over small penetrating artery ostia (also known as branch occlusive disease; Holmstedt et al., 2013).

In a large prospective study, different stroke mechanisms were studied in acute stroke patients with ICAS (>50%) as evaluated by MRI DWI (diffusion‐weighted imaging) and MRA, CTA, or DSA (Kim, Nah, et al., 2012). The following prevalence of stroke mechanisms was reported in intracranial atherosclerosis: artery‐to‐artery embolism (46%), perforator branch occlusion (21%), in situ thrombo‐occlusion (19%), hemodynamic impairment (1%), and mixed (13%; Wong et al., 2002). In another study of more than 130 symptomatic ICAS patients, similar results were shown: artery‐to‐artery embolism caused approximately 50% of strokes, perforator occlusion about 25%, hypoperfusion in <10%, and mixed in 16% (Lopez‐Cancio et al., 2014).

With regard to localization, intracranial atherosclerosis in the anterior circulation was more often associated with artery‐to‐artery embolism (52% vs. 34%) and less often with perforator branch occlusion (12% vs. 40%) than intracranial atherosclerosis in the posterior circulation (Kim, Nah, et al., 2012).

Determining whether intracranial artery stenosis is symptomatic or asymptomatic may not be straightforward, since at least 19% of recurrent strokes in ICAS could have been caused by other coexisting mechanisms such as cardioembolism and extracranial large artery disease (Famakin, Chimowitz, Lynn, Stern, & George, 2009). Moreover, studies with microembolic signal monitoring by transcranial Doppler indicate that a combined embolism‐hypoperfusion mechanism could be common in symptomatic MCA stenosis (Leung et al., 2015; Wong et al., 2002). In a prospective study of 30 patients with symptomatic MCA stenosis, TCD monitoring showed microembolic signals in eight out of 16 patients with border zone infarcts (Wong et al., 2002). Hemodynamic compromise in conjunction with multiple small artery‐to‐artery emboli may result in border zone infarctions because of failure to clear emboli in a poorly perfused brain area (Wong et al., 2002). Also, for stroke recurrence in ICAS, it was demonstrated that artery‐to‐artery thrombo‐embolism (as demonstrated with TCD) in combination with impaired washout at border zones (as demonstrated with watershed infarction on MRI DWI) was a common mechanism (Leung et al., 2015).

### 2.1 Imaging stroke mechanism in lacunar stroke

In patients with intracranial atherosclerosis, those with lacunar and nonlacunar presentations have similarly high risks of recurrent stroke (18% vs. 22% over a mean follow‐up of 1.8 years in the Warfarin‐Aspirin Symptomatic Intracranial Disease [WASID] trial) (Khan, Kasner, Lynn, & Chimowitz, 2012). The differentiation of subtypes of lacunar ischemic stroke caused by focal macroatheroma (known as branch occlusive disease) or fibrohyalinosis (attributed to hypertension, microatheroma and endothelial failure) could be important because patients without macroatheroma could get less long‐term benefit from antiatheromatous treatments (Benavente et al., 2012; Wardlaw, Smith, & Dichgans, 2013). On conventional imaging, the perforating arteriolar lumen and atheroma in the perforating vessel are difficult to identify. Recently, 7‐Tesla MRI has become available, which has the potential to improve our understanding of small vessel disease (SVD) by visualizing the vascular pathology itself as well as parenchymal markers which previously could only be examined postmortem (Benjamin, Viessmann, MacKinnon, Jezzard, & Markus, 2015). With 7‐Tesla MRA, differences in the lenticulostriate arteries of patients with SVD, hypertension, and previous stroke have been reported with a reduced number of branches compared with healthy controls (Benjamin et al., 2015). Direct visualization of the affected lenticulostriate artery in a specific lacunar infarct has also been performed in a few selected number of cases (Kang et al., 2012). Vessel wall imaging with 7‐Tesla MRI for small vessel disease is a promising technique since it can be used to visualize basal intracranial vessel wall disease which may be useful in determining the role of intracranial atheroma in the pathogenesis of lacunar infarction (Benjamin et al., 2015).

## 3 Prognostic Imaging Features of Intracranial Atherosclerosis

### 3.1 Vascular lumen and degree of stenosis

The method currently used in clinical practice to grade atherosclerosis is measuring the degree of stenosis expressed in percentage of the total vessel lumen. However, a seemingly normal vascular lumen in patients with intracranial atherosclerosis does not necessarily indicate a healthy vessel segment since the lumen can remain normal for a long period of time because of vascular remodeling (Glagov, Weisenberg, Zarins, Stankunavicius, & Kolettis, 1987). Positive (outward) remodeling and large enhancing plaques are associated with unstable plaque morphology, resulting in plaque rupture, which has been demonstrated with high‐resolution imaging for the embolic subtype of intracranial atherosclerotic disease (Ryoo, Lee, Cha, Jeon, & Bang, 2015). Therefore, simply measuring the vessel lumen as a measure of intracranial atherosclerosis might underestimate the thromboembolic complication risk of these patients (Ma et al., 2010). However, the WASID trial showed that the degree of luminal stenosis did affect the risk of stroke recurrence. At a median follow‐up of 1.8 years, risk of stroke in the territory of the stenotic artery was 11% for patients with 50–69% stenosis, 18% for patients with 70–79% stenosis, 30% for patients with 80–89% stenosis while 9% in the 90–99% stenosis group (Kasner et al., 2006).

The severity of ICAS is also related to stroke mechanism (Jiang et al., 2006). In 80 symptomatic ICAS patients, stroke with the branch occlusive disease subtype had a milder degree of stenosis in comparison with nonbranch occlusive disease subtypes of ICAS patients (41% vs. 75%) (Ryoo et al., 2015). Although the severity of ICAS is related to the risk of border zone infarction (Wong et al., 2002), the risk of branch occlusive stroke is not associated with the severity of ICAS stenosis (Lopez‐Cancio et al., 2014).

Next to reduction in the lumen diameter, length of the stenosis also seems relevant since the risk of ipsilateral ischemic stroke one year after stenting is 8% for lesions of ≤5 mm, 12% for lesions 5–10 mm in length, and 56% in patients with ICAS length of >10 mm (Mori, Fukuoka, Kazita, & Mori, 1998).

### 3.2 Collateral status

Most of the infarcts in patients with MCA stenosis are smaller than the large cortical infarcts caused by acute MCA occlusion. This can be explained by the gradual development of sufficient leptomeningeal anastomosis between anterior, posterior, and middle cerebral arteries in the process of atherosclerotic stenosis of the MCA (Wong et al., 2002). More severe stenoses generally exhibit greater degrees of compensatory collateral flow as measured with DSA (Liebeskind et al., 2012).

Observations in clinical trials have confirmed an important role for collateral circulation in averting stroke risk in ICAS. In a post hoc analysis of the WASID trial, two divergent patterns were noted in the association of collaterals with stroke risk based on the severity of luminal stenosis. Collaterals were assessed on DSA with the American Society of Interventional and Therapeutic Neuroradiology (ASITN)/Society of Interventional Radiology (SIR) Collateral Flow Grading System (Higashida et al., 2003) (See Box 1).

Box 1 **ASITN/SIR Grading system for collaterals with digital substraction angiography.**

1

**Table:** 

Collateral Grade	
0	No collaterals visible to the ischemic site
1	Slow collaterals to the periphery of the ischemic site with persistence of some of the defect
2	Rapid collaterals to the periphery of the ischemic site with persistence of some of the defect and to only a portion of the ischemic territory
3	Collaterals with slow but complete angiographic blood flow of the ischemic bed by the late venous phase
4	Complete and rapid collateral blood flow to the vascular bed in the entire ischemic territory by retrograde perfusion

Collaterals were subsequently categorized as none (grade 0), poor (grades 1 or 2), or good (grades 3 or 4). Good collaterals demonstrated a protective effect on averting territorial stroke risk in severe (≥70%) intracranial stenosis (no or poor collaterals vs. good, 30% vs. 5%). However, the presence of any collaterals in moderate stenosis (50–69%) did not have a protective effect (Liebeskind et al., 2012). Nevertheless, the authors conclude that isolated measures of the degree of stenosis in an artery may be inadequate for identifying hemodynamic or embolic significance (Liebeskind et al., 2012).

Angiographic analysis of the collateral circulation of patients enrolled in the Stenting and Aggressive Medical Management for preventing recurrent stroke in intracranial stenosis (SAMMPRIS) trial showed that none of the 117 patients with good collaterals (66 patients in the medical arm and 51 in the stent arm) had strokes within 30 days of enrolment. In contrast, recurrent stroke occurred in 5/25 (20%) in medically treated patients and in 11/46 (24%) of stented patients in the group with partial recanalization and poor collaterals (Liebeskind et al., 2012).

In a retrospective cohort study in which composite vascular assessment in intracranial atherosclerosis was used, good collateral compensation in patients with compromised antegrade flow was associated with a more favorable outcome (modified Rankin scale 0–2 at 3 months: 92 vs. 57%) (Lau et al., 2012). Collateral status is increasingly being used for patient selection in ICAS studies (Gonzalez et al., 2015a; Miao et al., 2015), but the exact value of collateral status in ICAS needs further study before it can be used for decision‐making in clinical practice.

### 3.3 Plaque components

Various components of atherosclerotic plaques might also be an important indicator of thromboembolic risk. Atherosclerotic plaques can be defined as being either stable or unstable. Unstable plaques are soft, lipid‐rich, and have an inflamed thin fibrous cap. These unstable plaques are more vulnerable to plaque disruption than collagen‐rich plaques with a thick fibrous cap, which are considered stable plaques (Drouet, 2002; Libby, 1995; Loree, Kamm, Stringfellow, & Lee, 1992).

For intracranial atherosclerosis, these plaque characteristics could determine the likelihood of future ischemic events suggesting that even mild intracranial stenosis of <50% might be clinically relevant in the presence of an unstable plaque. In 259 autopsies of patients with ischemic stroke, 43% had at least one intracranial plaque inducing luminal stenosis graded ≥30%, which was considered to be the cause of the stroke in 6% of cases; among these, 73% were severe stenoses and 27% were stenoses between 30% and 75%. The authors conclude that stenosis graded 30–75% could be associated with occurrence of ischemic stroke and therefore be of clinical significance (Mazighi et al., 2008). A recent study investigating large cerebral arteries from 15 autopsy cases with noncardioembolic brain infarcts, found that 20% of advanced atherosclerotic lesions (i.e., thin fibrous cap atheroma and fibrocalcific plaques) only showed stenosis of <40% of the vessel lumen (Gutierrez et al., 2015). These findings suggest that the degree of stenosis does not fully account for cerebral atherosclerosis burden. However, the exact relevance of these findings is yet to be determined since patients with ICAS of <50% were excluded from the randomized controlled ICAS trials (Chimowitz et al., 2005, 2011; Zaidat et al., 2015). Further characterization of plaque morphology in vivo, not only in patients with severe ICAS but also in patients with milder degree of stenosis will provide more insight into the mechanism leading to ischemic stroke (Ritz et al., 2014).

## 4 Treatment of Intracranial Atherosclerotic Stenosis

### 4.1 Medical management

Optimal treatment for symptomatic ICAS is still evolving (Gao et al., 2015; Holmstedt et al., 2013; Turan et al., 2014). The current treatment of patients with ischemic events attributable to intracranial stenosis is based on a combination of antiplatelet drugs and optimization of blood pressure and LDL cholesterol values through lifestyle modification and drug treatment (Qureshi & Caplan, 2014). In recent years, the preference for dual antiplatelet therapy in patients with symptomatic ICAS has increased (Turan et al., 2014). Support for aggressive medical management (i.e., intensive risk factor management and combined aspirin plus clopidogrel) comes from the lower early recurrent stroke rates in the SAMMPRIS trial (Derdeyn et al., 2014; Zaidat et al., 2015) compared with historical controls from the WASID trial taking either aspirin or warfarin (Chimowitz et al., 2005; Turan et al., 2014). After adjustment for difference in baseline characteristics, patients from the WASID trial had an almost twofold higher risk of the SAMMPRIS trial primary outcome (12.6% vs. 21.9% for any stroke or death within 30 days after enrolment or ischemic stroke in the territory of the qualifying artery beyond 30 days of enrolment). This finding supports the hypothesis that the lower rate of the primary outcome in the medical arm of SAMMPRIS compared with WASID patients was a result of the aggressive medical management used in the SAMMPRIS trial (Chaturvedi et al., 2015).

Further support for dual antiplatelet therapy comes from the CLAIR (Clopidogrel plus aspirin vs. aspirin alone for reducing embolization in patients with acute symptomatic cerebral or carotid artery stenosis) trial, which showed that combined aspirin and clopidogrel decreased the number of microembolic signals on transcranial Doppler ultrasound compared with aspirin alone in patients with symptomatic ICAS (31% vs. 54%) (Turan et al., 2014; Wong et al., 2010).

### 4.2 Stenting versus medical management

The SAMMPRIS trial has shown that patients with a transient ischemic attack or stroke due to 70–99% stenosis of a major intracranial artery had greater benefit from aggressive medical management alone than with percutaneous transluminal angioplasty and stenting with the Wingspan stent plus aggressive medical management (Derdeyn et al., 2014). Intracranial stenting was associated with an increased risk of recurrent ischemic events or death when compared with medical therapy in patients treated for symptomatic intracranial stenosis (15% vs. 6% at 30 days and 23% vs. 15% at 32 months follow‐up). In the VISSIT (the Vitesse Intracranial Stent Study for Ischemic Stroke Therapy) trial, worse outcomes after stenting were also shown with a different (i.e., balloon expanding) stent in comparison with medical therapy in symptomatic intracranial atherosclerotic stenosis (24% vs. 9% at 30 days, 36% vs. 15% at 12 months follow‐up) (Compter et al., 2015; Derdeyn et al., 2014; Zaidat et al., 2015). Despite the disappointing performance of intracranial stenting in both VISSIT and SAMMPRIS, it is possible that improvement of operator experience and periprocedural factors such as blood pressure management may lead to improved outcomes after stenting in future trials (Wang et al., 2014; Zaidat et al., 2015). Newer stent technology could possibly also enhance the safety and success of future endovascular procedures (Zaidat et al., 2015). Device selection based on arterial access and lesion morphology is currently implemented in a large Chinese study (Wang et al., 2014), in which self‐expanding stents are preferably used in patients with tortuous arterial access, while balloon‐expandable stents are preferred in patients with smoother access.

Currently, aggressive medical management remains the standard of care for patients with ICAS (Kernan et al., 2006). This is further supported by a recent study which showed that a majority of symptomatic high‐grade intracranial plaques regress or remain quiescent by 1 year under intensive medical therapy (Leung et al., 2015).

### 4.3 Treatment of high‐risk subgroups

It has been argued that a high‐risk subgroup of patients who do not respond to medical management might possibly benefit from stenting (Abou‐Chebl & Steinmetz, 2012; Gao et al., 2015). In previous trials (Compter et al., 2015; Derdeyn et al., 2014; Zaidat et al., 2015), patients were enrolled based on stenosis grade without further use of lesion‐based risk models or distinction between the different subtypes of ischemia (perforator occlusion, in situ thrombosis, hemodynamic stroke, and embolic stroke). Hemodynamic infarctions that relate to a low flow situation based on critical stenosis with insufficient leptomeningeal supply are from a pathophysiological point of view better suited for endovascular therapy than other ICAS stroke mechanisms (Lutsep et al., 2015). In a recent post hoc analysis of the SAMPRISS trial, a distinction was made between ICAS patients with and without hypoperfusion symptoms defined as symptoms related to change in position, effort on exertion, or recent change in antihypertensive (Lutsep et al., 2015). However, no additional radiological evidence of hypoperfusion was used for this subgroup analysis. For the 31 patients (14% of the total study population) who had a hypoperfusion symptom as a qualifying event, the 2‐year probability of an outcome event was 7.0% with medical treatment. For the 18 patients with hypoperfusion symptoms in the stent group, this 2‐year probability was 5.6%. For ICAS patients without hypoperfusion symptoms, these 2‐year probabilities were 15.1% in the medical group and 21.9% in the stent group. Since no statistical significance for a treatment interaction was reached, the authors conclude that there is no evidence to support the use of stenting in this subgroup of patients with hypoperfusion symptoms (Lutsep et al., 2015). However, in our view, a beneficial effect of stenting for patients with hypoperfusion symptoms cannot be ruled out either.

Since patients presenting with perforator ischemia possibly have an excessive risk of periprocedural stroke (due to occluding stenosed perforators by plaque shifting), these patients are excluded in a new multicenter randomized clinical trial (Gao et al., 2015). In a prospective registry study that evaluates stenting of symptomatic ICAS (Wang et al., 2014), imaging features are used for patient selection in addition to the degree of stenosis. For inclusion, hypoperfusion has to be demonstrated by means of reduced blood flow on CT perfusion or SPECT, poor collaterals on DSA, hemodynamic ischemic lesion on MRI, or high peak systolic velocities on TCD (Wang et al., 2014). The first results of intracranial stenting in patients with severe ICAS and radiological evidence of hemodynamic failure look promising, with a low rate of recurrent stroke or TIA (4% at 30 days follow‐up) and a high technical success rate (97%) (Miao et al., 2015). Whether preprocedural hemodynamic imaging (such as CT perfusion) to demonstrate hypoperfusion will result in a different long‐term outcome of intracranial stenting in comparison with aggressive medical therapy remains to be proven (Gao et al., 2015).

### 4.4 Other therapies

In the EC–IC (extracranial–intracranial) bypass trial, patients with intracranial stenotic lesions consistently had worse outcome following direct revascularization in comparison with medical therapy alone (The EC/IC Bypass Study Group, 1985). More than 700 ICAS patients were randomly assigned to the best medical care, and almost 700 ICAS patients to the same regimen with the addition of bypass surgery joining the superficial temporal artery and the middle cerebral artery. The patients were followed up for an average of 56 months. There was no evidence that surgery decreased the number of strokes; 18% of the medical patients and 20% of the surgical patients had one recurrent stroke. Two or more recurrent strokes occurred in 10% of the medical patients and in 11% of the surgical patients (The EC/IC Bypass Study Group, 1985).

Novel therapies that appear promising in symptomatic ICAS patients include remote limb ischemic conditioning (which involves producing repetitive, transient noninjurious ischemia of a limb that results in release of circulating mediators that may increase cerebral blood flow) (Chimowitz & Derdeyn, 2015; Meng et al., 2012) and indirect revascularization surgery called encephalo‐duro‐arterio‐synangiosis (EDAS) (Gonzalez et al., 2015a). In a recently published study, patients with symptomatic ICAS were considered for indirect revascularization by EDAS if they had TIAs or nondisabling stroke in the territory of the stenosis, despite optimal medical treatment. The rationale for EDAS in ICAS patients is that some patients with ICAS develop adequate leptomeningeal collaterals on their own, but those patients who remain symptomatic can potentially benefit from the additional collaterals created as a result of the EDAS procedure. If additional imaging selection criteria are used in ICAS (i.e., only inclusion of patients with evidence of hypoperfusion and poor collateral flow), surgical treatment with EDAS appears to reduce the rate of recurrent ischemic events (5.6%) at a median follow‐up of 2 years (Gonzalez et al., 2015b).

## 5 Imaging of Intracranial Atherosclerosis

In general, timely diagnosis is important in ICAS because time is a predictor of stroke recurrence, that is, a higher recurrent stroke risk in patients within 2.5 weeks after the first ischemic event than later (hazard ratio: 1.7; 95% CI 1.1–2.7) (Kasner et al., 2006). An important feature of intracranial atherosclerosis is that plaques are not fixed stenoses, but are dynamic and subject to remodeling under medical therapy (Famakin et al., 2009).

### 5.1 Radiologic mimics of intracranial atherosclerosis

While anatomic diagnosis of arterial narrowing is made with reasonable accuracy, ascribing the etiology for the stenosis remains challenging in clinical practice (Prabhakaran & Romano, 2012). Radiologic mimics of ICAS are often encountered such as vasculitis, moyamoya disease, fibromuscular dysplasia, vasospasm, radiation‐induced vasculopathy, or dissection and may require multimodal imaging to be able to distinguish between them (Prabhakaran & Romano, 2012). Luminal imaging techniques traditionally used for the assessment of vasculopathy do not adequately assess vessel wall pathology and can be of limited value in differentiating between causes of intracranial vasculopathies (Mossa‐Basha et al., 2015). Patterns and intensity of postgadolinium enhancement of vascular lesions with MRI T1 postcontrast vessel wall imaging have been used to differentiate between intracranial atherosclerosis and other vasculopathies. ICAS generally reveals eccentric thickening with variable enhancement, whereas vasculitis shows smooth, intense, and homogeneous enhancement; and reversible cerebral vasoconstriction syndrome (RCVS) has minimal to no enhancement and minimal wall thickening (Mossa‐Basha et al., 2015). A recent study with 7‐Tesla MRI (Dieleman et al., 2014a) has demonstrated that intracranial vessel wall abnormalities alone did not enable better differentiation between ischemic stroke etiologies. The results of intracranial vessel wall abnormalities should be combined with the infarct type (lacunar infarcts, smaller and larger macroinfarcts) for ascribing different stroke etiologies (Dieleman et al., 2014a).

### 5.2 Imaging modalities

#### 5.2.1 Digital subtraction angiography

**Figure 1:**
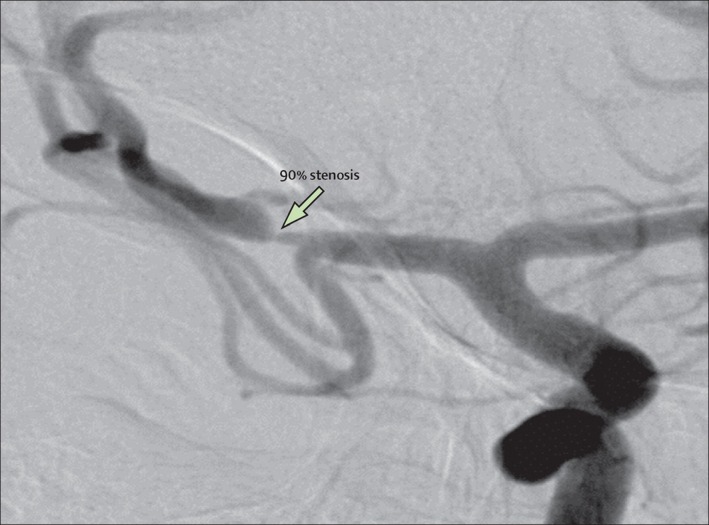
Catheter angiogram showing 90% stenosis of the right middle cerebral artery. “Reprinted from Holmstedt et al. (2013) with permission from Elsevier”

Digital subtraction angiography is considered the gold standard for quantification of stenosis and assessment of collateral flow (Kasner et al., 2006; Qureshi & Caplan, 2014). Such precise information is most valuable in patients in whom intracranial angioplasty, stenting, or EDAS is an option (Qureshi & Caplan, 2014). DSA has been used extensively to assess ICAS because of the inherent high spatial resolution leading to high imaging quality (Figure 1). However, disadvantages include high costs, limited availability, and a small risk (<1%) of serious periprocedural complications (Cloft, Joseph, & Dion, 1999; Cloft, Lynn, Feldmann, & Chimowitz, 2011; Willinsky et al., 2003). Although DSA is the criterion standard, it is usually not performed in the acute setting when diagnostic imaging in acute ischemic stroke patients is required. DSA is only used in the acute setting in selected patients when intraarterial recanalization procedures are indicated, and not for determining the presence of ICAS per se.

On two‐dimensional (2D) DSA, the severity of stenosis is quantified as a ratio between the wall diameter at the point of maximum narrowing and the reference diameter of the proximal normal artery (Samuels, Joseph, Lynn, Smith, & Chimowitz, 2000). Studies about intra‐ and extracranial stenosis suggest that 2D‐DSA may incorrectly estimate true stenosis when its findings are compared with those of other imaging modalities (such as 3D‐rotational angiography or high‐resolution CTA) as well as measurements in histological specimens (Netuka et al., 2010; Safain et al., 2014). Vessel stenosis due to plaque occurs in three dimensions and with 2D‐DSA these spatial features are flattened. Measuring a 3D phenomenon such as stenosis on a 2D imaging modality likely has inherent error (Safain et al., 2014; Streifler et al., 1994). A recent study with 18 patients with intracranial atherosclerosis has shown superior performance of high‐resolution CT angiography over 2D‐DSA and 3D‐rotational angiography in characterization of plaque morphology (such as ulceration, calcification, dissection of the plaque, and core protrusion into the vessel lumen). Compared to high‐resolution CTA, such lesions are missed in 61% (*n *= 11) when evaluation of intracranial atherosclerosis is done with 2D‐DSA, and in 50% of patients (*n *= 9) with 3D‐rotational angiography (Safain et al., 2014).

Digital subtraction angiography can also provide valuable information about collateral flow. In a recently published prospective study in a Chinese population, patient selection for intracranial stent placement was based on the presence of hemodynamic failure as demonstrated on DSA with an ASITN/SIR collateral score (See Box 1) of <3. This selection of patients for hemodynamic failure in ICAS resulted in a <5% 30‐day rate of stroke, TIA, or death after intracranial stenting (Miao et al., 2015), substantially lower than the 15% and 24% 30‐day rates in the SAMMPRIS and VISSIT trials (Chimowitz et al., 2011; Zaidat et al., 2015). Finally, DSA is the most accurate modality to evaluate ICAS after stent placement because CTA and MRA are limited by stent‐generated artifacts.

### 5.3 Transcranial Doppler ultrasound

Transcranial Doppler ultrasound is safe, inexpensive, and easily applied in clinical practice (Markus, 1999). Transcranial Doppler ultrasound provides real‐time flow information, can detect microembolic signals, and can provide information about cerebral autoregulation (Topcuoglu, 2012). However, its use in clinical practice is limited by a high operator dependency and the requirement of suitable temporal bone acoustic windows (which can be absent in up to 20% of patients) (Alexandrov, Demchuk, Wein, & Grotta, 1999; Markus, 1999; Meseguer et al., 2010; Seidel, Kaps, & Gerriets, 1995).

One early study, including 130 acute ischemic stroke patients, reported a sensitivity of 87.5%, specificity of 88.6%, positive predictive value (PPV) of 87.5%, and a negative predictive value (NPV) of 88.6% of TCD for the detection of stenosis or occlusion compared with DSA (Alexandrov et al., 1999). In the prospective Stroke Outcomes and Neuroimaging of Intracranial Atherosclerosis (SONIA) trial, in which 407 ICAS subjects were enrolled who had experienced a transient ischemic attack or stroke in the prior 90 days, TCD ultrasound had a PPV of 36% and a NPV of 86% for the detection of 50–99% stenosis when compared with DSA (Feldmann et al., 2007). This study was limited to stenosis only and excluded vessel occlusions, probably explaining the lower reported PPV.

Transcranial color‐coded duplex ultrasound (TCCS), a technique that combines flow velocity measurements with imaging of parenchymal structures (Zipper & Stolz, 2002), overcomes this problem partially, but it would still require confirmatory testing with DSA. Recent studies using TCCS, reported a sensitivity of 72.9–88.9%, specificity of 82.9–94.8%, PPV of 51.1–79.4%, and NPV of 77.3–99.3% for the identification of stenosis or occlusion (Hou et al., 2009; Roubec et al., 2011). However, these ultrasound techniques are not often used in clinical practice for evaluation of ICAS because of the high operator dependency.

### 5.4 Noncontrast CT

In general, the initial imaging study in stroke patients is noncontrast CT (NCCT). Calcification of coronary arteries as measured on NCCT has been strongly associated with the risk of future cardiac events (Criqui et al., 2014). Multiple studies aimed to determine a similar correlation between calcification in the intracranial ICA and ischemic stroke (Bos et al., 2012; Chung et al., 2010). Although calcification did not predict ischemic stroke, the degree of calcification in the intracranial ICA as assessed with NCCT was related to stenosis as shown with DSA (Sohn, Cheon, Jeon, & Kang, 2004; Taoka et al., 2006; Woodcock, Goldstein, Kallmes, Cloft, & Phillips, 1999). Calcification volume measured on NCCT appeared to be a risk factor for stroke (Bos et al., 2014). However, a direct causal link between intracranial calcification and ischemic stroke has not yet been proven (Chimowitz & Caplan, 2014). Another major disadvantage of measuring calcification on NCCT is that this imaging modality cannot assess the distal intracranial arteries, since they are too small and only large calcified plaques can be detected (Sohn et al., 2004). Measuring calcification on NCCT might therefore probably be not enough to provide insight into intracranial atherosclerosis and the subsequent stroke risk.

### 5.5 CT angiography

CT angiography is a useful screening tool because it is only minimally invasive, fast, and more widely available in clinical practice than MRA and DSA. CT angiography has a high interoperator reliability when assessing stenosis grade (Bash et al., 2005) and it is less susceptible to motion artifacts compared with MRI techniques (Bash et al., 2005; Nguyen‐Huynh et al., 2008). Disadvantages are the exposure to radiation and the necessity of iodinated contrast material use, which may lead to allergic reaction and nephropathy in some cases (Bash et al., 2005). Computed tomography angiography is not suitable for depiction of vessels with a diameter smaller than 0.7 mm (Skutta, Furst, Eilers, Ferbert, & Kuhn, 1999; Villablanca et al., 2007), due to limited spatial resolution. Concern with branching arteries of <2 mm exists since slight differences in measurements might lead to a large difference in final estimation of degree of stenosis (Nguyen‐Huynh et al., 2008). Also, dense and extensive mural calcifications may reduce the accuracy of measuring the degree of stenosis with CTA (Marquering, Nederkoorn, Bleeker, van den Berg, & Majoie, 2013). Several studies reported problems of CTA imaging with the vertebral artery and the ICA in the region of the skull base (Graf, Skutta, Kuhn, & Ferbert, 2000; Skutta et al., 1999). However, studies with more advanced CTA postprocessing techniques have shown the possibility to reliably depict vessels close to the skull base (Bash et al., 2005; Johnson, Heath, Kuszyk, & Fishman, 1996; Kuszyk, Heath, Johnson, Eng, & Fishman, 1999; Kuszyk et al., 1995).

An overview of studies comparing CTA with DSA for identification of intracranial stenosis is presented in Table 1. Overall, studies show that CTA is useful as a screening tool for identification of ICAS and intracranial occlusion. It may therefore be used to exclude cases of ICAS, replacing the use of DSA in many cases. However, since some studies report a lower PPV of CTA compared with DSA, these authors suggest that DSA might be required for confirmation of CTA findings (Duffis et al., 2013; Graf et al., 2000; Liebeskind, Kosinski, Saver, & Feldmann, 2014; Nguyen‐Huynh et al., 2008; Roubec et al., 2011).

**Table 1:** Overview of studies reporting test characteristics for ICAS using CTA compared with DSA

Authors and years	Population	No.	Design	Reference standard	Stenosis cut‐off, %	Sensitivity, %	Specificity, %	PPV, %	NPV, %
Skutta et al. (1999)	Stroke/TIA, suspected aneurysms	112	Retrospective	DSA	70–99	78.0	–	81.8	–
					30–69	61.0	–	84.6	–
					10–29	66.0	–	28.0	–
					0–9	99.5	–	99.0	–
Bash et al. (2005)	Stroke/TIA	28	Retrospective	DSA	30–99	98	98	78	100
Nguyen‐Huynh et al. (2008)	Stroke/TIA	41	Retrospective	DSA	50–99	97.1	99.5	–	99.8
Roubec et al. (2011)	Stroke/TIA	67	Retrospective	DSA	<50 and 50–99	81.5	98.7	78.6	98.6
Duffis et al. (2013)	Stroke/TIA	57	Retrospective	DSA	50–99	96.6	99.4	94.9	99.6
Liebeskind et al. (2014)^a^	Stroke/TIA	20	Prospective	DSA	50–99	–	–	46.7	73.0
					70–99	–	–	13.3	83.8

a Stenosis on CTA followed by occlusion on DSA was reported as a false positive. Occlusion was interpreted as absence of stenosis. A negative CTA followed by occlusion on DSA was scored as a true negative.

### 5.6 MR angiography

#### 5.6.1 Time‐of‐flight MRA

**Figure 2:**
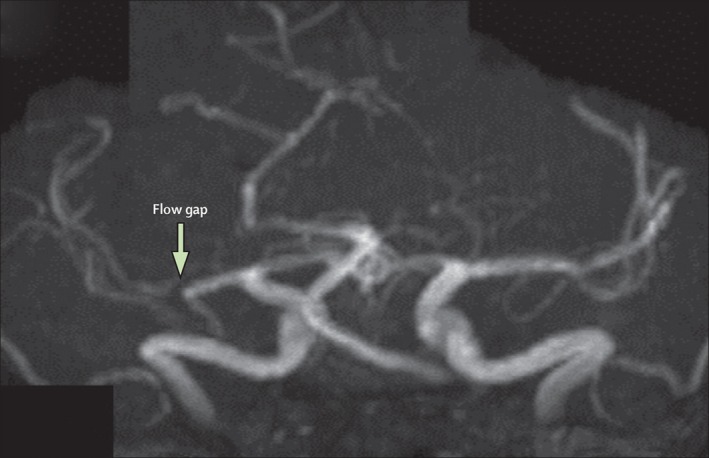
Magnetic resonance angiography showing a flow gap in the right middle cerebral artery in a patient with a recent right hemisphere infarct. This gap suggests a flow‐limiting stenosis, but the degree of stenosis cannot be accurately measured “Reprinted from Holmstedt et al. (2013) with permission from Elsevier”

Time‐of‐flight (TOF) MRA is a noninvasive technique that does not use any radiation nor contrast material to visualize arteries (Nederkoorn et al., 2002) (Figure 2). The main disadvantage is the high susceptibility of TOF‐MRA to flow‐related artifacts. Complete absence of MR signal in an artery can occur even though the vessel is not entirely occluded. Severe stenosis not only causes these flow‐related artifacts in most cases but also in <70% stenosis these artifacts are reported (Nederkoorn et al., 2002), which influences the reported sensitivity and specificity values of MRA compared with DSA (Table 2).

**Table 2:** Overview of studies on test characteristics of TOF‐MRA and CE‐MRA compared with DSA for identification of ICAS

Authors and years	Population	No.	Modality	Design	Reference standard	Stenosis cut‐off, %	Group subdivision:	Sensitivity, %	Specificity, %	PPV, %	NPV, %
Korogi et al. (1994)	Suspected stenosis	131	TOF‐MRA	Retrospective	DSA	>50^a^	ICA	85	96	–	–
							MCA	88	97	–	–
Stock et al. (1995)	Multiple^b^	50	TOF‐MRA	Prospective	DSA	>50	Carotid, basilar	86	86	–	–
Furst et al. (1996)	Stroke/TIA	70	TOF‐MRA	Prospective	DSA	<30 and 30–99^a^	None	–	99	–	–
Hirai et al. (2002)	Suspected stenosis	18	TOF‐MRA	Prospective	DSA	>50^a^	None	92	91	–	–
Bash et al. (2005)	Stroke/TIA	28	TOF‐MRA	Retrospective	DSA	30–99	None	70	99	63	98
Choi et al. (2007)	Suspected stenosis	39	TOF‐MRA	Retrospective	DSA	50–99	None	78–85	95	75–79	95–97
Sadikin et al. (2007)	Confirmed stenosis	45	TOF‐MRA	Retrospective	DSA	>29	None	94	96	84	99
						>49	None	95	96	70	99
Feldmann et al. (2007)	Suspected stenosis	407	TOF‐MRA	Prospective	DSA	50–99	None	–	–	59	91
Wutke et al. (2002)	Suspected stenosis	30	CE‐MRA	Retrospective	DSA	70–99	ICA bifurcation	100	92	89	100
							ICA distal	100	90	25	100
							MCA	–c	98	–c	100
							ACA	100	84	11	100
Nederkoorn et al. (2003)	Suspected stenosis	51	CE‐MRA	Prospective	DSA	70–99	ICA	90–91	76–77	–	–
Willinek et al. (2005)	Suspected stenosis	50	CE‐MRA	Prospective	DSA	70–99	All supraaortic arteries	100	99	94	100
							ICA	100	97	95	100

a Values include occlusion.

b Population of neurologic deficits and other intracranial problems and malformations, seven stenoses found.

c No cases with high‐grade stenosis in the MCA present in population.

Postprocessing techniques have been optimized throughout the years and MR scanners with increasingly higher magnetic field strengths (and thus increased spatial resolution) have been developed for use in clinical practice (Korogi et al., 1997; Willinek et al., 2004). These optimized techniques are thought to underlie the improved ability of TOF‐MRA to detect intracranial stenosis. Early studies with 1.5 T TOF‐MRA compared with DSA showed sensitivity values ranging from 85% to 88% and specificity values from 86% to 99% for the detection of steno‐occlusive disease (Furst et al., 1996; Korogi et al., 1994; Stock, Radue, Jacob, Bao, & Steinbrich, 1995). Although more recent studies report improved values of TOF‐MRA compared with DSA in the detection of intracranial stenosis, other studies failed to show this. These discrepancies result in varying reported test characteristics, with sensitivity values ranging from 70% to 95%, specificity values from 95% to 99%, PPV from 59% to 84%, and NPV from 91% to 99% (Bash et al., 2005; Choi et al., 2007; Feldmann et al., 2007; Sadikin et al., 2007). Even with newer postprocessing techniques, DSA is still required occasionally to confirm TOF‐MRA findings (Sadikin et al., 2007).

Studies comparing TOF‐MRA and CTA for its ability to identify intracranial stenosis and occlusion compared with DSA conclude that CTA is superior to TOF‐MRA; CTA had higher sensitivity than MRA (98% compared with 70%) and a higher PPV than MRA (93% compared with 65%) (Bash et al., 2005).

The combination of CTA and MRA for detection of intracranial stenosis and occlusion compared with DSA has also been studied. Although MRA alone had a sensitivity of 92% and a specificity of 91% for the detection of stenosis ≥50%, adding CTA increased these values to 100% and 99%, respectively, and resulted in a predictive value of 93% (Hirai et al., 2002). These are promising results for the identification of ICAS in ischemic stroke patients, indicating that a combination of different modalities could be able to replace DSA in the imaging of ICAS. However, more research is needed to validate the combined use of CTA and MRA in clinical practice.

#### 5.6.2 Contrast‐enhanced MRA

Limited spatial resolution and sensitivity for correct timing of imaging after contrast administrations have been described as disadvantages of contrast‐enhanced (CE) MRA (Wutke et al., 2002). Techniques to time the arrival of contrast are used to optimize the quality of CE MRA. Spatial resolution of CE MRA has been improved with newer techniques, including more efficient coil systems. Studies with these improved techniques report a sensitivity and specificity ranging from 90% to 100% and 76% to 99%, respectively (Nederkoorn et al., 2003; Willinek et al., 2005; Wutke et al., 2002). These values do not seem to indicate an advantage of CE MRA over optimized TOF‐MRA (Nederkoorn et al., 2003). An overview of available values for TOF‐MRA and CE‐MRA compared with DSA is presented in Table 2. At this moment, studies do not indicate that MRA can replace the gold standard DSA with respect to the degree of vessel stenosis in the intracranial vasculature.

## 6 Novel Techniques

More detailed visualization of intracranial vessels is possible with advanced technological developments, for example, increased spatial resolution and improved postprocessing techniques. With high‐resolution imaging, the focus is shifted toward more detailed pathological characterization of intracranial atherosclerosis, in addition to measurements of the vessel lumen (Arenillas, 2011; Chen, Wong, Lam, Zhao, & Ng, 2008).

### 6.1 High‐resolution computed tomography angiography

A new CTA technique called ultra‐high resolution cone‐beam CTA (CB‐CTA) has been used for the evaluation of intracranial atherosclerotic stenosis. CBCT‐A is an invasive catheter‐based modality with a radiation dose similar to that of conventional CTA. CB‐CTA did not differ significantly in assessing the absolute percent stenosis of lesions when compared with other 3D modalities such as 3D rotational angiography (Safain et al., 2014). However, both CB‐CTA and 3D rotational angiography differed in the measurement of percent stenosis when compared with traditional 2D‐DSA (Safain et al., 2014). Since intracranial stenosis is a three‐dimensional phenomenon, it is possible that CB‐CTA and 3D rotational angiography are more accurate than standard 2D‐DSA. However, these findings from a single study (Safain et al., 2014) need further validation with DSA in a prospective manner.

In addition, CB‐CTA provided greater resolution when compared with DSA. New information about plaque morphology was identified in more than 60% of patients when CB‐CTA was used following standard DSA (Damaskos, Aartman, Tsiklakis, van der Stelt, & Berkhout, 2015; Safain et al., 2014). Whether the plaque morphology evaluated on CB‐CTA has any additional benefit in comparison with conventional CTA and MRA still needs to be proven.

### 6.2 High‐resolution MRI

**Figure 3:**
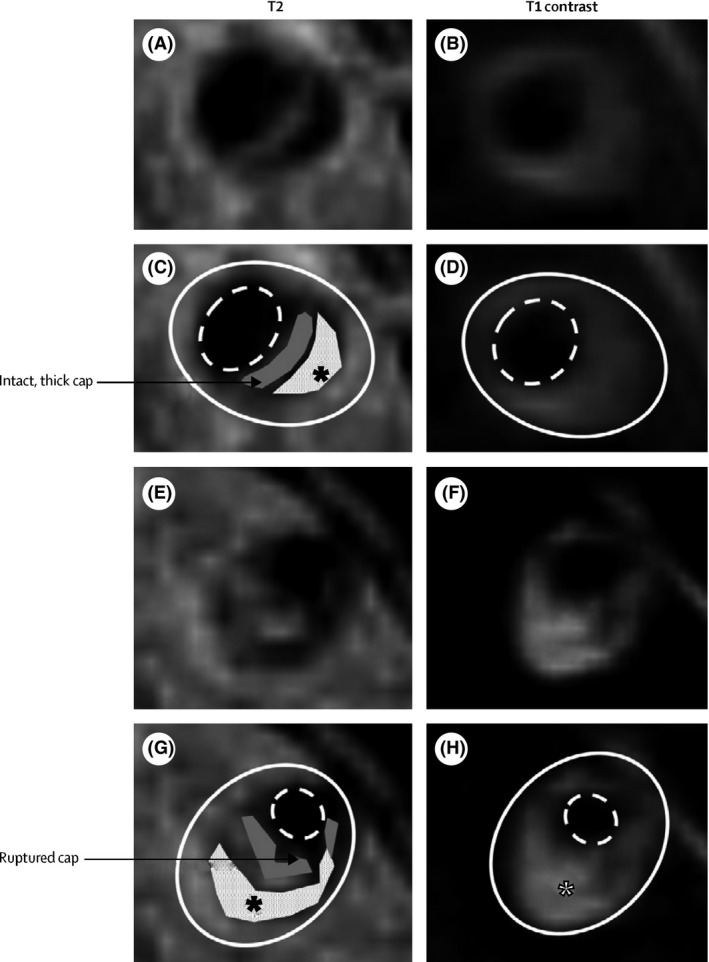
High‐resolution MRI of vertebral artery stenoses with plaque components Panels A–D show T2‐weighted and T1 postcontrast images (panels C and D have plaque components marked) of a crosssection of a vertebral artery plaque with a thick, intact, fibrous cap (gray) and lipid core (white with black asterisk). Panels E–H show T2‐weighted and T1 postcontrast images (panels G and H have plaque components marked) of a cross section of a vertebral artery plaque with a ruptured fibrous cap (gray) and lipid core (white with black asterisk), which enhances with contrast (white asterisk) and is also indicative of plaque rupture. The solid white line shows the outside vessel wall and the dashed white line the lumen “Reprinted from Holmstedt et al. (2013) with permission from Elsevier”

High‐resolution (HR) MRI can already be accomplished at a field strength of 1.5‐Tesla (T) (Klein et al., 2010; Natori et al., 2014). Studies with higher magnetic field strengths (3T or 7T) report the ability to detect smaller intracranial arteries, the vessel wall, and atherosclerotic plaques (Chung, Kwak, Hwang, & Jin, 2012; Chung et al., 2014; Dieleman et al., 2014b; Kim, Lim, et al., 2012; Li et al., 2009; Niizuma, Shimizu, Takada, & Tominaga, 2008; Ryu, Jahng, Kim, Choi, & Yang, 2009; Xu et al., 2010; Zhu et al., 2013) (Figure 3). The stenosis grade for the HR MRI has a good agreement with the DSA stenosis grade (Ryu et al., 2009).

An important advantage of HR MRI is that various components from the plaque can be identified, in which the presence of lipid core and intraplaque hemorrhage has been associated with vulnerability of atherosclerotic plaques. In carotid atherosclerotic disease, a large lipid core can be identified with HR MRI as an isointense or slightly hyperintense signal on T1‐weighted MR imaging and hypointense signal on T2‐weighted MR imaging. Calcification shows a hypointense signal intensity on all MRI sequences, whereas intraplaque hemorrhage shows a hyperintense signal on T1‐weighted images (Chu et al., 2004; Chung et al., 2014; Saam et al., 2005; Yoshida et al., 2008).

Promising results of intracranial vessel wall imaging have been published recently, in which 7T MRI was compared with histopathology. Areas of foamy macrophages were generally seen as proton attenuation‐, T2‐, and T2*‐ hypointense areas, whereas areas of increased collagen content showed more ambiguous signal intensities (van der Kolk, Zwanenburg, Denswil, et al., 2015). Several HR MRI studies described differences between symptomatic and asymptomatic patients with intracranial atherosclerotic plaques, thereby identifying plaque characteristics that are probably related to the development of symptoms (Chung et al., 2012; Ryu et al., 2009; Xu et al., 2010). Hyperintense foci on T1‐ and/or T2‐weighted MRI were more frequently seen in symptomatic MCA plaques than in asymptomatic plaques (Ryu et al., 2009); these foci might therefore represent the lipid core or intraplaque hemorrhage. Additional features from HR MRI studies have shown prognostic value such as plaque burden (Ryu et al., 2009; Xu et al., 2010; Zhao et al., 2015), plaque location (Xu et al., 2011; Zhao et al., 2015), remodeling pattern (Chung et al., 2012; Xu et al., 2010; Zhao et al., 2015), and vessel wall enhancement pattern (Lou, Ma, Ma, & Jiang, 2013; Skarpathiotakis, Mandell, Swartz, Tomlinson, & Mikulis, 2013; Vakil et al., 2013). Recently, different plaque characteristics have been demonstrated for different etiologies of ICAS. Plaque enhancement was observed in all but one of the nonbranch occlusive disease patients, while enhancement was seen in only one‐fourth of branch occlusive disease patients. Enhancement was more frequently distributed in the branch occlusive disease group on the side where the perforators arose. Moreover, ICAS patients with branch occlusive disease showed a characteristic remodeling pattern (less outward) than nonbranch occlusive disease ICAS patients (Ryoo et al., 2015).

Further prospective studies with larger sample sizes are needed to confirm these promising results of HR MRI in intracranial atherosclerosis in recent studies.

## 7 Conclusions

Currently, aggressive medical management remains the standard of care for patients with ICAS. Noninvasive imaging modalities are useful screening tools for ICAS. Use of advanced MRA and CTA techniques has improved the assessment of the intracranial vasculature, with high sensitivity and specificity values for the identification of one element of ICAS, degree of vessel stenosis, as compared with DSA. However, consensus about the optimal noninvasive imaging strategy to identify and evaluate ICAS has not been established so far.

Newer techniques, using high‐resolution imaging with either CT or MR techniques, are currently the subject of extensive research. Although further validation is needed, it appears that HR MRI is likely the most promising technique so far to achieve detailed assessment of intracranial atherosclerosis imaging.

### 7.1 Directions for future research

Further characterization of prognostic radiological features as well as differentiation of hemodynamic, embolic, and perforator lesion patterns could play an important role in patient selection and outcome in currently ongoing and future clinical trials. New trials are also necessary to assess the optimal therapy in patients with symptomatic ICAS and recurrent strokes in the setting of failed aggressive medical therapy. Identification of stroke mechanisms of intracranial atherosclerosis in relation to future ischemic events, could well lead to the identification of a high‐risk subgroup of ICAS patients who might benefit from more aggressive treatment approaches.

Currently available imaging modalities should be evaluated on specific advantages of each technique rather than focusing exclusively on the severity of the intracranial stenosis. Newer imaging techniques, using high‐resolution imaging, should be validated for identification of high‐risk patients who are likely to fail medical therapy. The role of plaque characteristics and hemodynamic features need to be fully appreciated in future treatment studies.

## Funding Information

Dutch Heart Foundation (Grant/Award Number: ‘2011T055’) Netherlands Organisation for Scientific Research (Grant/Award Number: ‘ZonMW‐Veni’)

## Conflicts of Interest

None.
